# Alterations in whole-brain dynamic functional stability during memory tasks under dexmedetomidine sedation

**DOI:** 10.3389/fneur.2022.928389

**Published:** 2022-10-28

**Authors:** Lin-Lin Liu, Jian-Long He, Vivian Man-Ying Yuen, Xuebing Xu, Xuan Guan, Yan Qiu, Yingzi Wang, Chao-Jun Jian, Zhibo Wen, Ke-Xuan Liu

**Affiliations:** ^1^Department of Anesthesiology, Nanfang Hospital, Southern Medical University, Guangzhou, China; ^2^Department of Anesthesiology, The University of Hong Kong–Shenzhen Hospital, Shenzhen, China; ^3^Department of Radiology, Zhujiang Hospital, Southern Medical University, Guangzhou, China; ^4^Radiology Center, Department of Medical Imaging, The University of Hong Kong–Shenzhen Hospital, Shenzhen, China; ^5^Department of Anesthesiology and Perioperative Medicine, Hong Kong Children's Hospital, Hong Kong, Hong Kong SAR, China

**Keywords:** dexmedetomidine, memory, functional neuroimaging, magnetic resonance imaging, sedation, anesthetics

## Abstract

**Purpose:**

This study aimed to explore the neurological effects of dexmedetomidine-induced sedation on memory using functional stability, a whole-brain voxel-wise dynamic functional connectivity approach.

**Methods:**

A total of 16 participants (10 men) underwent auditory memory task-related fMRI in the awake state and under dexmedetomidine sedation. Explicit and implicit memory tests were conducted 4 h after ceasing dexmedetomidine administration. One-sample Wilcoxon signed rank test was applied to determine the formation of explicit and implicit memory in the two states. Functional stability was calculated and compared voxel-wise between the awake and sedated states. The association between functional stability and memory performance was also assessed.

**Results:**

In the awake baseline tests, explicit and implicit memory scores were significantly different from zero (*p* < 0.05). In the tests under sedation, explicit and implicit memory scores were not significantly different from zero. Compared to that at wakeful baseline, functional stability during light sedation was reduced in the medial prefrontal cortex, left angular gyrus, and right hippocampus (all clusters, *p* < 0.05, GRF-corrected), whereas the left superior temporal gyrus exhibited higher functional stability (cluster *p* < 0.05, GRF-corrected). No significant associations were observed between functional stability and memory test scores.

**Conclusions:**

The distribution and patterns of alterations in functional stability during sedation illustrate the modulation of functional architecture by dexmedetomidine from a dynamic perspective. Our findings provide novel insight into the dynamic brain functional networks underlying consciousness and memory in humans.

## Introduction

Dexmedetomidine is widely employed for clinical sedation and general anesthesia, as it induces and maintains a natural state of non-rapid eye movement sleep and has minimal effects on respiration (1). It is also a safe and reliable medication for non-painful procedural sedation and preoperative sedation in children (2). Although dexmedetomidine is increasingly used for procedural sedation, there is a paucity of studies reporting its effects on memory formation. Preservation of intraoperative implicit or explicit memory may influence a patient's subsequent emotions and behavior in similar future encounters, which may contribute to negative emotions or behavior (3). Nevertheless, the relationship between memory encoding and brain functional responses during dexmedetomidine sedation remains unclear.

Dexmedetomidine is commonly used for procedural sedation in non-painful diagnostic radiographic studies, which enables the more accurate investigations of the relationship between drug-induced sedation and memory function in clinical settings. Both task-based and resting-state functional magnetic resonance imaging (fMRI) have been applied to explore the neurobiological effects of dexmedetomidine and other sedatives. Previous fMRI studies of sedatives have predominantly relied on time-averaged methods assuming static patterns of brain functional connectivity across time (4, 5). However, several studies have provided evidence for dynamic, condition-dependent characteristics of brain activity, highlighting the importance of temporal dynamic analyses (6, 7). Although dynamic integration and coordination to external and internal stimuli are critical for brain activity, maintaining stable and constant connectivity patterns is also essential for memory formation and consciousness (8, 9). Prior studies have explored dynamic functional stability by comparing between-state functional connectivity across several resting-state fMRI sessions (10, 11). Recently, Li et al. (12) successfully demonstrated a robust and reliable analysis of within-state functional stability characteristics in higher-order association and unimodal regions by measuring the concordance of whole-brain voxel-wise dynamic functional connectivity (DFC). This dynamic stability approach has the potential to provide novel insight into the mechanisms underlying memory function in dexmedetomidine-induced sedation.

This study aimed to explore the neurological effects of dexmedetomidine-induced sedation on memory using functional stability, a whole-brain voxel-wise dynamic functional connectivity approach. To investigate the association between brain function and sedation effect, we applied functional stability analysis to fMRI data of a stable auditory memory task before and during dexmedetomidine-induced light sedation. We hypothesized that amnesia effects would be associated with the dexmedetomidine-induced suppression of functional stability in certain brain regions.

## Methods

### Ethics and study population

This study was approved by the Medical Ethics Committee of the University of Hong Kong–Shenzhen Hospital (Approval No: Ethics [2018]110) on August 3, 2018. Written informed consent was obtained from all participants prior to their inclusion in the study. The trial was registered prior to patient enrollment at www.chictr.org.cn (ChiCTR1800018324; Principal investigator: Lin-Lin Liu; Date of registration: September 11, 2018). This manuscript adheres to the applicable CONSORT guidelines.

Twenty participants were originally recruited from the Anesthesiology Clinic at the University of Hong Kong-Shenzhen Hospital from August 2019 to December 2020. Inclusion criteria were as follows: native Chinese speakers, aged between 18 and 40 years, right hand-dominant, American Society of Anesthesiologists physical status scores of I or II, an education level of junior high school or higher, and normal cognition as indicated by the Mini-Mental State Examination. Exclusion criteria included hearing impairments, language disorders, obstructive sleep apnea syndrome, contraindications for MRI examination (e.g., metal implants in the skull or claustrophobia), neurological or psychiatric conditions, structural brain abnormalities, and excessive head motion during fMRI scan. Overall, four participants were excluded according to the exclusion criteria (one claustrophobia, one structural brain abnormality, and two excessive head motion). Finally, sixteen participants (10 men; median age = 32.0 years, range: 25–36) were included in the study.

### Auditory memory task and dexmedetomidine administration

Participants were allowed to settle in a 3 T MRI system (Skyra; Siemens Healthineers, Erlangen, Germany) with nasal cannula oxygen supply (2 L/min) and intravenous access. Each participant's head was positioned in a 20-channel integrated head and neck coil and stabilized using a sponge and MRI-compatible headphones. Participants were instructed to close their eyes and listen to a set of 20 Chinese words presented during the awake state and after dexmedetomidine sedation. Participants were informed that their recognition and exclusion performance of words heard during the two sessions would be evaluated 4 h after scanning. Two of the three Chinese word lists were randomly selected and delivered *via* an integrated visual and audio stimulation system for fMRI (SA-9939, Sinorad, Shenzhen, China) with MRI-compatible headphones. One of the lists was played during the first session when participants were awake to allow conscious auditory verbal learning.

After completion of the first list, dexmedetomidine was administered at a loading dose of 0.5 μg/kg for 10 min *via* an infusion pump, followed by a continuous infusion of 0.5 μg/kg/h to maintain sedation. End-tidal carbon dioxide gas, pulse oximetry, non-invasive blood pressure cuff, and electrocardiogram monitoring were acquired using an MRI-compatible physical monitor. The desired level of sedation was assessed by our anesthesiologists using the Observer's Assessment of Alertness/Sedation Rating Scale (OAAS) (13). Once a target sedation depth of OAAS score of 3–4 was achieved and maintained, the other randomly selected study word lists were played to allow auditory learning during sedation. Dexmedetomidine administration was ceased immediately after the fMRI scans in the sedation session.

The above-mentioned study lists, test lists, and audio recordings for participants were created as previously described by Tian et al. (14) (see Supplementary materials, Section Memory test paradigm). In brief, 100 non-polysyllabic Chinese two-character words were selected from the Chinese dictionary. Then a validation test was performed on 10 undergraduate participants (five men) to identify 60 two-character words from the list with a formation frequency of 17–67% using word stem completion method. Those 60 Chinese characters were assigned to three study lists with 20 words each (seven concrete words and 13 abstract words). Regarding study list recordings, a word was presented in 3 s (1.5 s reading plus 1.5 s silence), then an auditory stimulus period was constructed for 60 s (20 words × 3 s). Each study list was repeated 10 times, which made the length of the entire recording to be 10 min (60 s × 10). Two test lists were created with the stems of 10 two-character words from each of the three study lists with identical average formation frequencies. Each test list was recorded once with an interval of 10 s between two characters. For each participant, two of the three study lists were randomly selected for auditory verbal learning in two states, while the third one was used as the distractor list in the memory test to determine the participants' basic hit rate. Inclusion and exclusion tests were conducted 4 h after cessation of dexmedetomidine administration. Explicit and implicit memory scores were calculated as described by Jacoby et al. (15) (see Supplementary materials, Section Memory test paradigm). The study design is illustrated in Supplementary Figure 1.

### MRI data collection

fMRI was performed using motion-corrected T2^*^-weighted gradient echoplanar images (64 × 64 matrix; 3 × 3-mm in-plane resolution; slice thickness, 5 mm; 35 slices; axial acquisition; repetition time (TR), 2,000 ms; echo time (TE), 30 ms; flip angle, 90°). High-resolution T1-weighted anatomical images (three-dimensional magnetization-prepared rapid acquisition gradient echo recalled sequence; 256 × 256 matrix; 1 mm^3^ voxels; 176 slices; sagittal acquisition; TR, 2,300 ms; TE, 3.43 ms; flip angle, 9°) were acquired prior to fMRI scans. Each session commenced with five dummy scans to ensure steady-state magnetization. The study lists served as an auditory stimulus, including an auditory period of 60 s (20 words × 3 s). Each of the study lists was repeatedly played 10 times when the participant was awake and under sedation, and fMRI scanning was performed simultaneously. Thus, two 10-min fMRI sessions were performed, in which 305 functional brain volumes were acquired with a TR of 2 s/volume.

### FMRI data preprocessing

Imaging data were processed using Statistical Parametric Mapping (SPM12, http://www.fil.ion.ucl.ac.uk/spm/) software package and the Data Processing and Analysis for Brain Image (DPABI, http://rfmri.org/DPABI) toolbox (16). The first five dummy scan volumes were deleted. The remaining functional images were subjected to standard slice-timing correction and realignment. In head motion assessments, all fMRI data were within 3 mm of displacement or 3° of rotation. Frame-wise displacement (FD) calculated using Jenkinson's formula (17) was applied for additional thresholding of head motion. Nuisance covariates including linear drift, head motion parameters based on the Friston-24 model, volumes with FD > 0.5, white matter signals, and cerebrospinal fluid signals were regressed out from the data. Subsequently, the structural images were co-registered with the mean functional image, segmented, and normalized to the Montreal Neurological Institute (MNI) space by diffeomorphic anatomical registration through the exponentiated Lie algebra (DARTEL) (18). The normalized functional images were then band-pass-filtered with a frequency of 0.01–0.1 Hz, resampled into 3-mm cubic voxels, and smoothed with an isotropic 6-mm full-width half-maximum (FWHM) Gaussian kernel.

### Calculation of functional stability

Whole-brain voxel-wise functional stability was calculated as described by Li et al. (12) Pearson's correlation coefficients between the time course of each voxel and all other voxels confined in the gray matter mask in consecutive time windows of data were calculated using a sliding-window approach, with a window length of 64 s (32 TRs) and sliding step of 4 s (19). This resulted in a series of DFC maps across the 135 time windows. The functional stability of each voxel was calculated using Kendall's coefficient of concordance (KCC, also referred to as Kendall's W) of DFC maps with time windows as raters based on the following equation:


$$\begin{array}{lll} W & = & \frac{12S}{K^{2}\left(N^{3}-N\right)}\\ S & = & {\overset{N}{\underset{n=1}{\sum }}R_{n}^{2}-\frac{1}{N}\left(\overset{N}{\underset{n=1}{\sum }}R_{n}\right)}^{2} \end{array}$$

where K is the number of windows, N is the number of connections of a given voxel with all voxels within the mask, and R_n_ is the sum of the rank for the nth connection across all windows. W (range, 0–1) quantified the stability of the DFC of a given voxel. Specifically, the gray matter mask was created by thresholding the mean gray matter density at 0.2, covering all cortical regions and subcortical nuclei, and intersected with a group mask with 90% coverage of functional images for both sessions. Z-standardization across the gray matter mask was applied to the KCC results to enable comparison across states and participants (20). For a given voxel or region, a higher KCC value is indicative of dynamic functional architecture that is more stable or lacks flexibility over time, depending on its intrinsic role.

Window length is a key parameter that reflects spontaneous brain activity dynamics, i.e., dynamic functional stability results are sensitive to the choice of sliding window length. To verify our findings, functional stability was further calculated using three other combinations of window size and sliding step (window size = 64 s and sliding step = 2 s; window size = 50 s and sliding step = 4 s; window size = 80 s and sliding step = 4 s), and the between-state pairwise comparisons were repeated.

### Statistical analysis

Statistical analysis was performed using GraphPad Prism version 8 for Windows (GraphPad Software, San Diego, CA, USA, www.graphpad.com). Demographic data and memory test results are presented as means [standard deviation (SD)] for continuous variables, or medians [interquartile range (IQR)] as proportions for categorical variables. A one-sample Wilcoxon signed rank test was used to determine whether the explicit and implicit memory scores were significantly different from zero. The significance level α was set as 0.05; i.e., *p* < 0.05 was considered to indicate statistical significance.

fMRI metrics were compared using the DPABI toolbox. One-sample *t*-tests were performed on the KCC z-scores of the two sessions to generate the *t*-value spatial distribution maps. KCC z-scores were compared between the two sessions using a voxel-based paired-sample *t*-test, with age, sex, and head motion (mean FD) as covariates. Correction for multiple comparisons was performed by applying a strict criterion of Gaussian random field (GRF) theory to the results, constituting a permutation test with threshold-free cluster enhancement (TFCE) with a threshold of p < 0.05 (21). Mean functional stability values of each significant cluster were extracted as regions of interest (ROIs) for pairwise comparisons. We further investigated the correlations between functional stability differences and memory test performance in the awake state and after sedation using Spearman's correlation analysis.

## Results

### Demographic and memory test data

Demographic information including age, sex, weight, body mass index (BMI), dexmedetomidine dose for achieving the target sedation depth, and OAA/S score is presented in Table 1. In the awake baseline tests, explicit [40.0 (12.5–67.5)] and implicit [6.7 (1.7–28.5)] memory scores were significantly different from zero. In the tests under sedation, explicit [10.0 (0–10.0)] and implicit [−2.5 (−13.8–3.0)] memory scores were not significantly different from zero. Hence, explicit and implicit memory formation was observed at baseline but not during dexmedetomidine sedation. The memory test scores are presented in Table 2.

**Table 1:** Demographic data of participants (*n* = 16).

**Parameter**	**Statistic**	**Value**
Age (y)	mean (SD)	30.9 (3.7)
Weight (kg)	mean (SD)	65.0 (12.8)
Sex, male	N (percent)	10 (63)
BMI	mean (SD)	22.8 (3.4)
Dexmedetomidine dose (μg.kg^−1^)	Median IQR (range)	0.58 0.04 (0.54, 0.69)
OAA/S score	Median IQR (range)	30 (3, 4)

IQR, interquartile range; OAA/S, the Observer's Assessment of Alertness/Sedation Rating Scale.

**Table 2:** Explicit and implicit memory scores before and after dexmedetomidine sedation.

**Session**	**Memory test**	**Value** **(median, IQR)**	**P**
Awake	Explicit	^*^40.0 (12.5, 67.5)	0.001
	Implicit	^*^6.7 (1.7, 28.5)	0.023
Sedation	Explicit	10.0 (0, 10.0)	0.059
	Implicit	−2.5 (−13.8, 3.0)	0.095

* p ≤ 0.05, compared with zero.

### Task-related functional stability characteristics and correlational analysis

**Figure 1:**
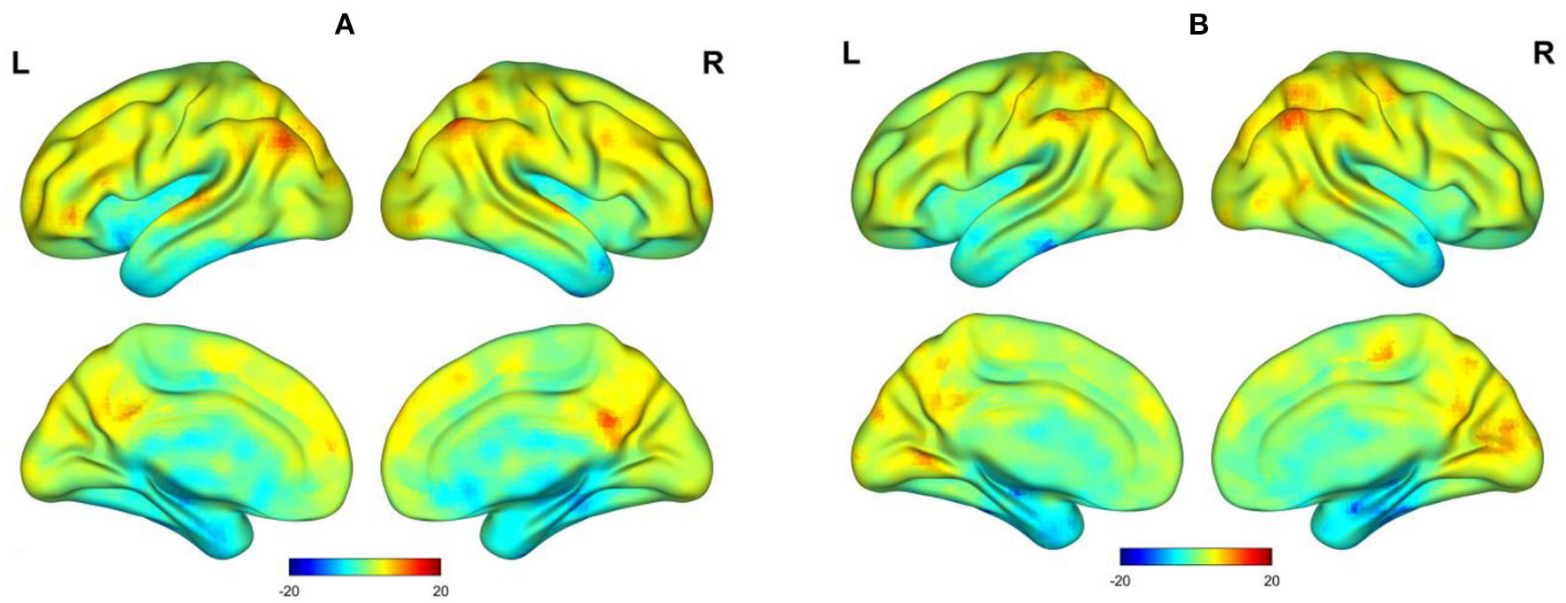
Surface view of *t*-value spatial distribution maps of one-sample *t*-tests on functional stability (z-scores) in an auditory memory task. **(A)** Awake state. **(B)** Dexmedetomidine sedation state.

The spatial distribution of the functional stabilities of the two states is depicted in Figure 1, which were mapped using BrainNet Viewer (http://www.nitrc.org/projects/bnv/) (22) for 3D visualization. During the awake scan, higher functional stability was observed predominantly in the bilateral angular gyrus (AG), posterior cingulate cortex (PCC)/precuneus, lateral prefrontal cortex (lPFC), left medial prefrontal cortex (L-mPFC), and superior temporal gyrus (STG). During light sedation, brain regions with higher functional stability included the bilateral AG, calcarine sulcus, PCC/precuneus, and postcentral gyrus. In contrast, similar brain regions with lower functional stability in both states were observed, including the anterior temporal lobe (ATL), hippocampus, orbitofrontal cortex (OFC), and insula.

**Figure 2:**
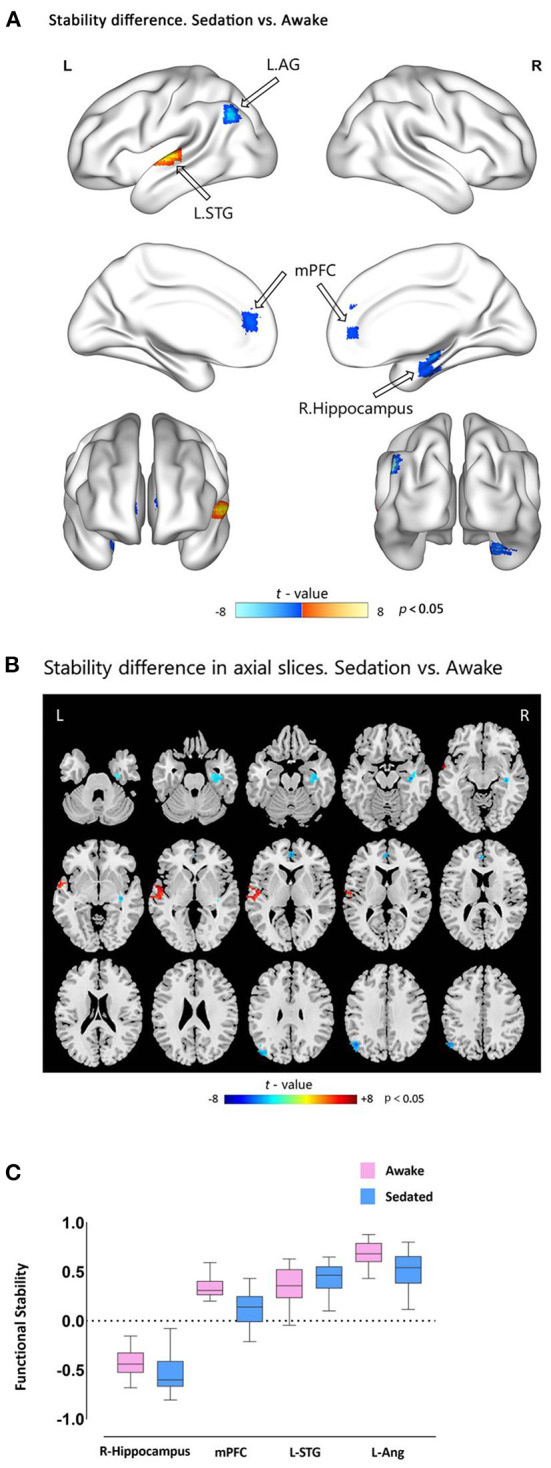
Functional stability differences between awake and dexmedetomidine sedation states. **(A)** Brain maps of *t*-values showing the results of paired-sample *t*-tests comparing functional stability during sedation vs. awake states. Cold-color clusters represent brain regions with lower stability during sedation than during the awake state (indicated by hollow arrows), and vice versa. **(B)** The axial slices view of the above mentioned *t*-test results. **(C)** Box plots showing the functional stability differences in the significant clusters between the two states. mPFC, medial prefrontal cortex; AG, angular gyrus; STG, superior temporal gyrus.

Comparison of light sedation to wakeful baseline revealed that functional stability was reduced in the mPFC, left AG, and right hippocampus (all clusters, *p* < 0.05, GRF-corrected), whereas the left STG (cluster *p* < 0.05, GRF-corrected) exhibited elevated functional stability (Figure 2 and Table 3). These findings were successfully reproduced using functional stability calculated from three other combinations of sliding window size and step, indicating that the results of the functional stability analysis were not significantly affected by sliding window options (Supplementary Figures 2–4). As indicated in Supplementary Table 1, no significant correlations were observed between functional stability and explicit or implicit memory scores.

**Table 3:** Stability difference of awake vs. dexmedetomidine sedation state.

**Brain regions**	**Cluster size** **(voxels)**	**MNI coordinates**			**Peak** ***t*-value**
		**X**	**Y**	**Z**	
mPFC	43	0	48	6	−5.8518
L.AG	18	−48	−66	36	−7.1781
R.Hippocampus	59	39	−15	−18	−5.9789
L.STG	87	−66	−9	3	7.9992

mPFC, medial prefrontal cortex; AG, angular gyrus; STG, superior temporal gyrus.

## Discussion

This study employed a temporal dynamic approach to quantify brain functional stability during dexmedetomidine-induced sedation. Functional stability was decreased in the mPFC, left AG, and right hippocampus, and increased in the left STG after dexmedetomidine sedation relative to baseline, accompanied by explicit and implicit memory suppression. These findings expand our knowledge of the mechanisms underlying the effects of sedative agents on memory function from a temporal dynamic perspective.

Maintaining stable functional organization patterns during dynamic processing is an essential feature of consciousness (23). Unimodal regions underscoring primary sensorimotor processing have fewer functional connections, whereas association regions involved in complex cognitive functions have more widespread connections and typically function as core hubs (10, 11). These regions continuously coordinate and integrate information at multiple time points and states *via* distributed connections. Indeed, stable functional connectivity modes are necessary for maintaining consciousness and memory formation (8, 9). Thus, we hypothesized that dexmedetomidine would modulate functional stability in different brain regions, accompanied by changes in memory function.

Previous studies have reported higher stability in higher-order association regions and lower stability in unimodal primary regions from both static and dynamic perspectives, which were more prominent during task modulation (12, 24). The current results complement previous findings by comparing functional stability between awake (fully functional) and sedated (dysfunctional) states. Dexmedetomidine-induced sedation reduced stability in the mPFC, left AG, and right hippocampus, which are higher-order association hubs of the default mode network (DMN) and limbic network (LN) (25). Conversely, the left STG within the primary auditory cortex exhibited increased functional stability during sedation. These functional stability patterns provide a dynamic overview of the modulation of brain functional organization by sedative agents. Dexmedetomidine predominantly affected higher-order regions, in line with previous reports highlighting the susceptibility of integrative brain functions to modulation by sedative agents (26). Given their widely distributed functional connectivity, higher-order regions play critical roles in complex functions such as cognition and consciousness. Guldenmund et al. (27) reported reduced medial PCC-thalamic functional connectivity during dexmedetomidine-induced unresponsiveness. Golkowski et al. (28) used a sliding window approach similar to the approach used herein to investigate the temporal dynamics of between-network connectivity during propofol and sevoflurane anesthesia. They concluded that higher-order brain regions were crucial for the generation and transition of specific between-network connectivity patterns, irrespective of the anesthetic agent. Our findings suggest that the sedative effects of dexmedetomidine are underpinned by an impaired ability to maintain stable integration and communication patterns in higher-order regions.

A major finding of our study was that dexmedetomidine sedation attenuated functional stability in DMN regions, accompanied by impaired memory function. The DMN is a critical higher-order association network that plays key roles in internally directed thought, consciousness, and memory (29, 30). Our findings suggest that during sedation, DMN cortical hubs could not stably coordinate and integrate information, which may impair memory function. In a DFC study by Luppi et al. (31), integration was reduced in posterior DMN regions during propofol-induced unconsciousness, in agreement with our results.

Reduced functional stability was observed in the right hippocampus during dexmedetomidine sedation. Stability maps revealed lower functional stability in subcortical regions and regions close to the cavities/ventricles, including the ATL, OFC, hippocampus, and insula. These regions have higher susceptibility artifacts and lower signal-to-noise ratios (32), leading to decreased functional stability. Accordingly, caution should be exercised when interpreting these findings. Although hippocampus-related findings were confirmed in validation tests of multiple sliding window combinations, further studies using field-map corrections are warranted.

We observed elevated functional stability in left STG during sedation. As functional stability alterations are underpinned by the role(s) of a given brain region, higher functional stability may not necessarily indicate higher-order functions, and lower functional stability may not always represent functional impairments. The STG is a component of unimodal regions, whose neural activity is driven by external stimuli and regulation by higher-order regions (33). Compared to higher-order regions, unimodal regions process information in seconds rather than minutes (34), consequently requiring frequent reorganization of connections and leading to higher functional flexibility or low functional stability in task-related states. The elevated functional stability in the primary auditory cortex observed herein may be due to loss of flexibility under sedation. It is well-established that auditory words are perceived but not necessarily processed during sedation (35, 36). The neural mechanisms underlying this phenomenon may involve anesthesia-induced interruption of auditory processing *via* primary auditory cortex deactivation (37), blockade of sensory information pathways to higher-order processing networks (35), or disruption of higher-order cortico-thalamic networks (38). Our findings suggest that anesthetic agents induce increased rigidity in primary auditory cortex dynamics, potentially perturbing memory formation.

Our study has several limitations. First, we did not confirm a correlation between brain regions with functional stability alterations and memory test scores. This could be due to the small sample size, which limited statistical power. Larger sample sizes are required to detect subtler brain-behavior associations. Second, we did not compare light and deep sedation states, as per other studies using propofol. However, dexmedetomidine induces natural non-rapid sleep as a sedative effect (39). Hence, participants were sensitive to MRI-associated acoustic noise, and inducing deep sedation with dexmedetomidine irrespective of dosage was challenging. Third, functional stability results may be affected by smoothing options in terms of kernel parameters and smoothing order (24). The smoothing options used herein were identical to those in the initial study reporting functional stability calculations. Future studies should apply multiple smoothing options in validation tests. Finally, we did not apply surface-based analysis to functional stability calculations. Although surface-based analysis permits better registration, smoothing, and consequently higher precision compared to traditional volume-based analysis (40), these analyses require complex calculations, making application exceedingly difficult.

In conclusion, we demonstrated that dexmedetomidine disrupted functional stability in higher-order association brain regions and unimodal primary auditory cortex, accompanied by suppression of explicit and implicit auditory memory. The patterns of distribution and alterations in functional stability during sedation illustrate the modulation of brain functional architecture by sedative agents from a functional dynamic perspective. Imaging the temporal dynamic patterns of functional connectivity during sedation may yield further insight into dynamic brain functional networks underscoring consciousness and memory in humans.

## Data availability statement

The original contributions presented in the study are included in the article/Supplementary material, further inquiries can be directed to the corresponding authors.

## Ethics statement

The studies involving human participants were reviewed and approved by the Medical Ethics Committee of the University of Hong Kong–Shenzhen Hospital. The patients/participants provided their written informed consent to participate in this study.

## Author contributions

L-LL and J-LH: conceptualization and study design, analysis and interpretation of data, and writing up and finalizing the manuscript. VY: conceptualization, review, and editing of the manuscript. XX: acquisition of data, review, and editing of the manuscript. XG, YQ, YW, and C-JJ: patient recruitment, acquisition of data, and editing of the manuscript. ZW and K-XL: study design, supervision, review, and editing of the manuscript. All authors contributed to the article and approved the submitted version.

## Funding

This study was funded by the Science, Technology, and Innovation Commission of Shenzhen Municipality (No. JCYJ20160429190235638), Shenzhen Healthcare Research Project (No. 201601036), and High Level-Hospital Program, Health Commission of Guangdong Province, China (No. HKUSZH201901005).

## Conflict of interest

The authors declare that the research was conducted in the absence of any commercial or financial relationships that could be construed as a potential conflict of interest.

## Publisher's note

All claims expressed in this article are solely those of the authors and do not necessarily represent those of their affiliated organizations, or those of the publisher, the editors and the reviewers. Any product that may be evaluated in this article, or claim that may be made by its manufacturer, is not guaranteed or endorsed by the publisher.
