# Supplementary material for: Alterations in whole-brain dynamic functional stability during memory tasks under dexmedetomidine sedation
Source: Front Neurol. 2022 Oct 28;13:928389. doi: 10.3389/fneur.2022.928389 (PMC9650205; doi:10.3389/fneur.2022.928389)
Supplement: Supplementary file 1 [file Data_Sheet_1.docx]

Supplementary Material

**Section 1. Memory Test Paradigm**

The study lists, test lists are as follows:

Study list 1

高兴 美丽 特别 **运动 低头 年龄 道理** 严肃 失去 感觉

推动 用途 **金色** 生活 投资 追求 **车辆** 情感 **脱衣** 新鲜

Study list 2

展开 **明天 图书** 意思 **交通** 欢笑 **退出** 遗憾 智慧 偏执

**物理** **招手** 平凡 探索 虚心 组合 **风景** 人潮 养生 争气

Study list 3

**夜晚** 前面 学习 **武功** **老师** 知道 强大 战斗 **主人** **亲人**

信任 直接 **上下** **通知** **银行** 光明 飘扬 神奇 外出 热闹

Test list 1（Stem）

高 展 夜 美 明 前 低 交 老 道

退 强 严 遗 战 感 偏 亲 金 平

上 车 风 飘 情 人 神 脱 养 外

Test list 2（Stem）

特 运 年 失 推 用 生 投 追 新

图 意 欢 智 物 招 探 虚 组 争

学 武 知 主 信 直 通 银 光 热

Four hours after ceasing dexmedetomidine administration, a memory test consisting of inclusion and exclusion tests was conducted. Participants were asked to perform stem completion of the words they heard during the experimental process. Explicit and implicit memory scores were calculated based on the results of the two tests mentioned above using the equations of Jacoby et al.15 as follows: Explicit memory score = inclusion test score-exclusion test score

Implicit memory score = (exclusion test score)/((1-explicit memory score))-distractor score

The distractor score is the average of the inclusion and exclusion test scores when the distractor list was used for testing.


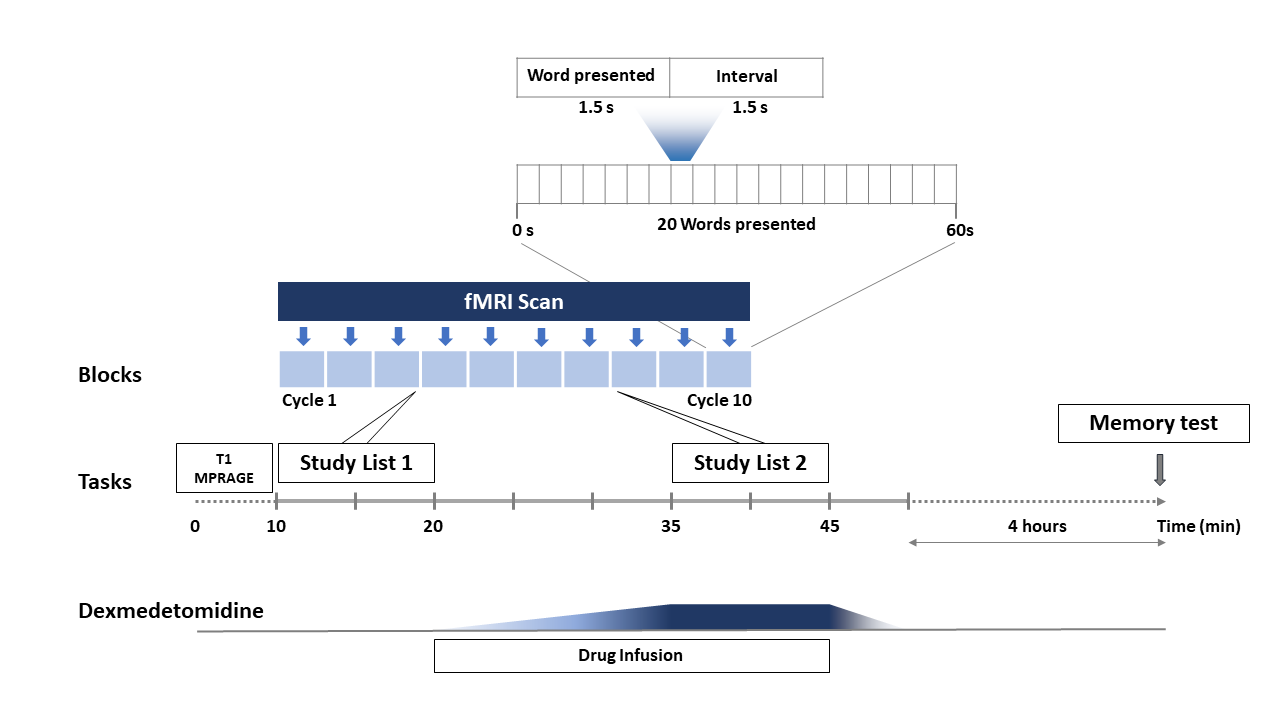


**Figure S1**. Experimental paradigm. Details of the auditory stimulus block, the task sequence, and the timing of dexmedetomidine infusion are shown in the top, middle, and bottom sections, respectively. Two auditory stimulus tasks were conducted before and during dexmedetomidine sedation, each consisting of 10 cycles of words. Each cycle consisted of 20 words. Abbreviation: fMRI, functional magnetic resonance imaging.

**Section 2. Validation Test Results**


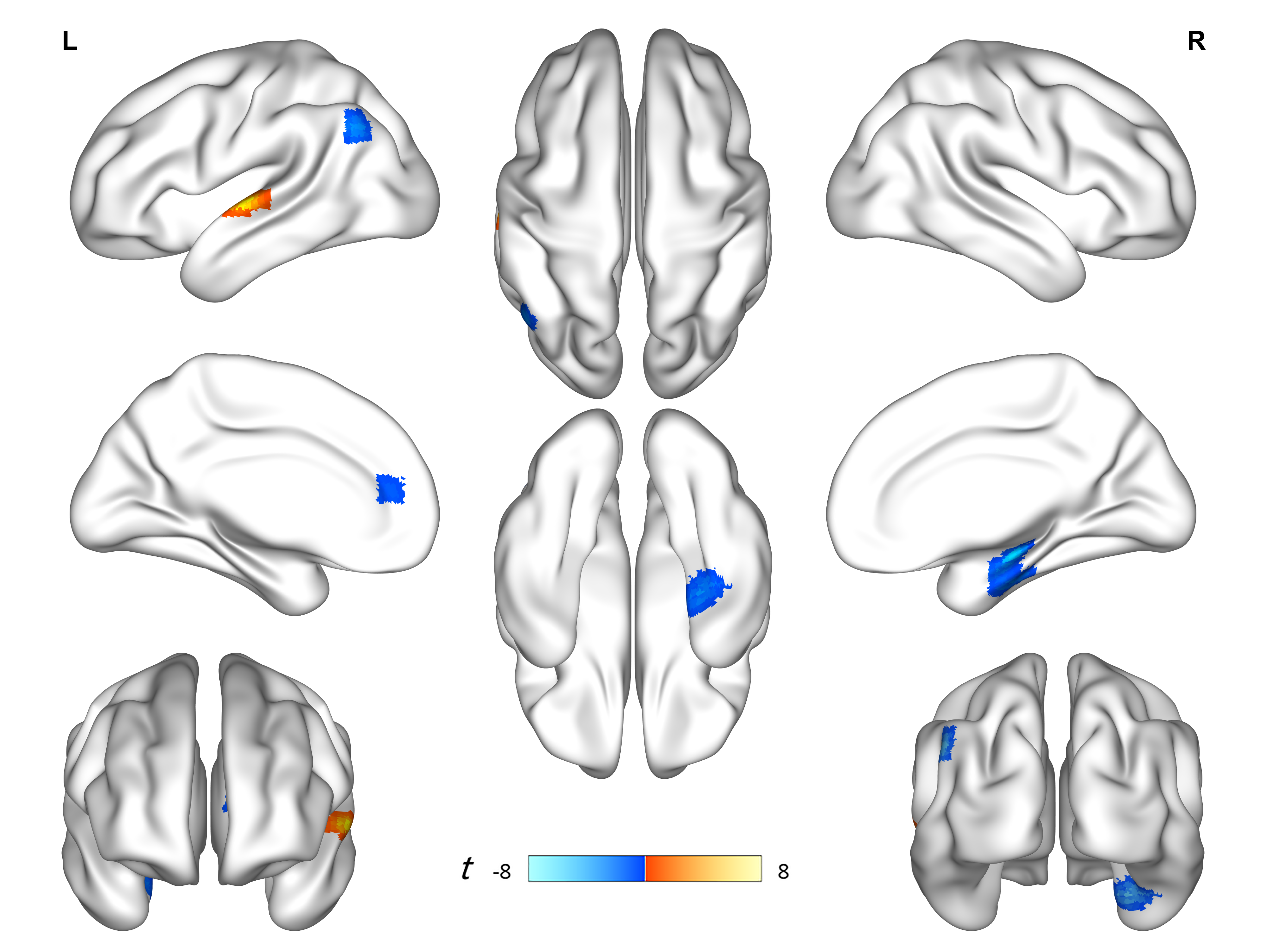


**Fig. S2.** Whole brain voxel-wise functional stability comparison between sedation and awake state, which is calculated from a combination of window size = 64 s and sliding step = 2 s. Abbreviations: L, left; R, right.


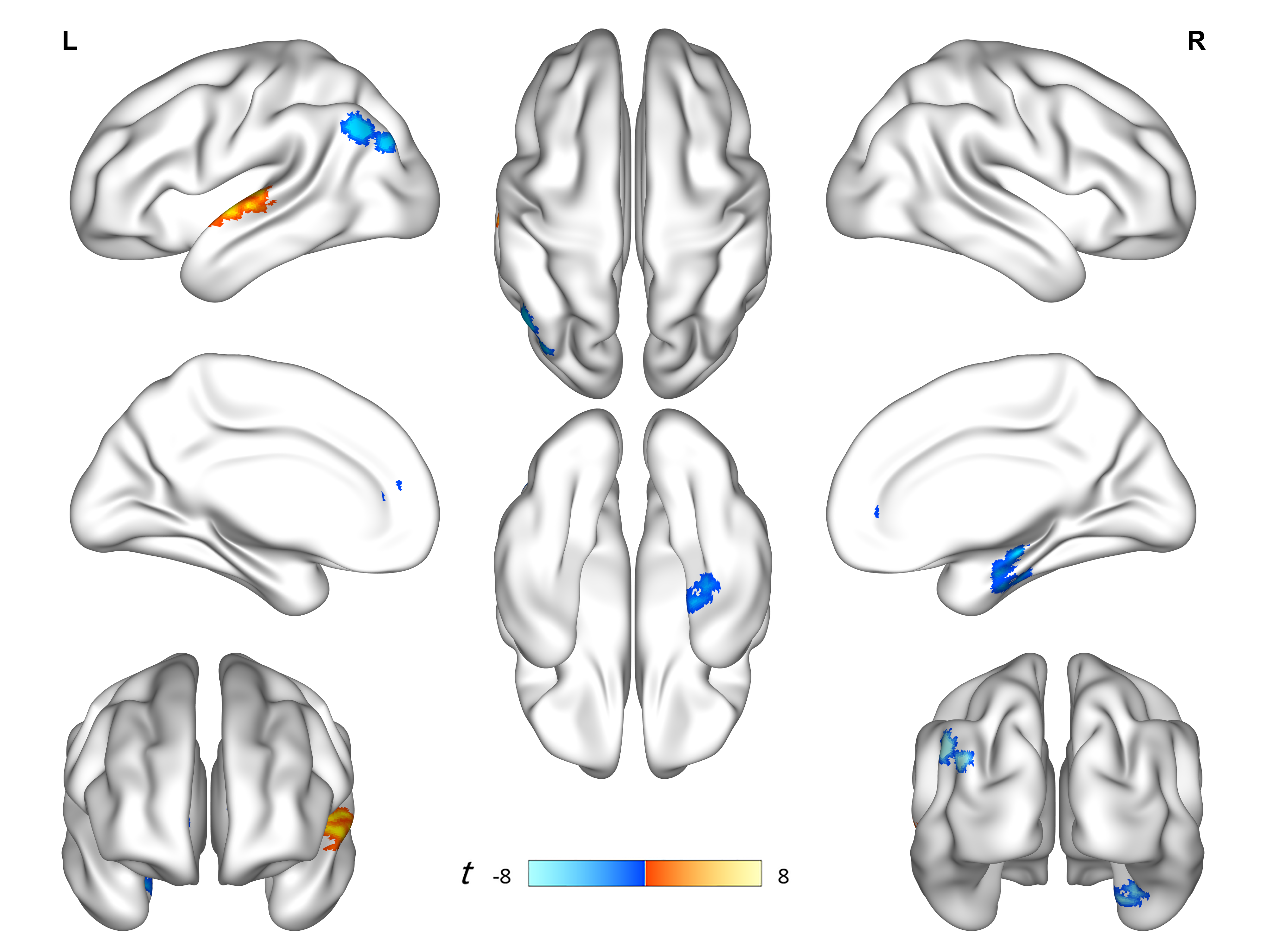


**Fig. S3.** Whole brain voxel-wise functional stability comparison between sedation and awake state, which is calculated from a combination of window size = 50 s and sliding step = 4 s. Abbreviations: L, left; R, right.


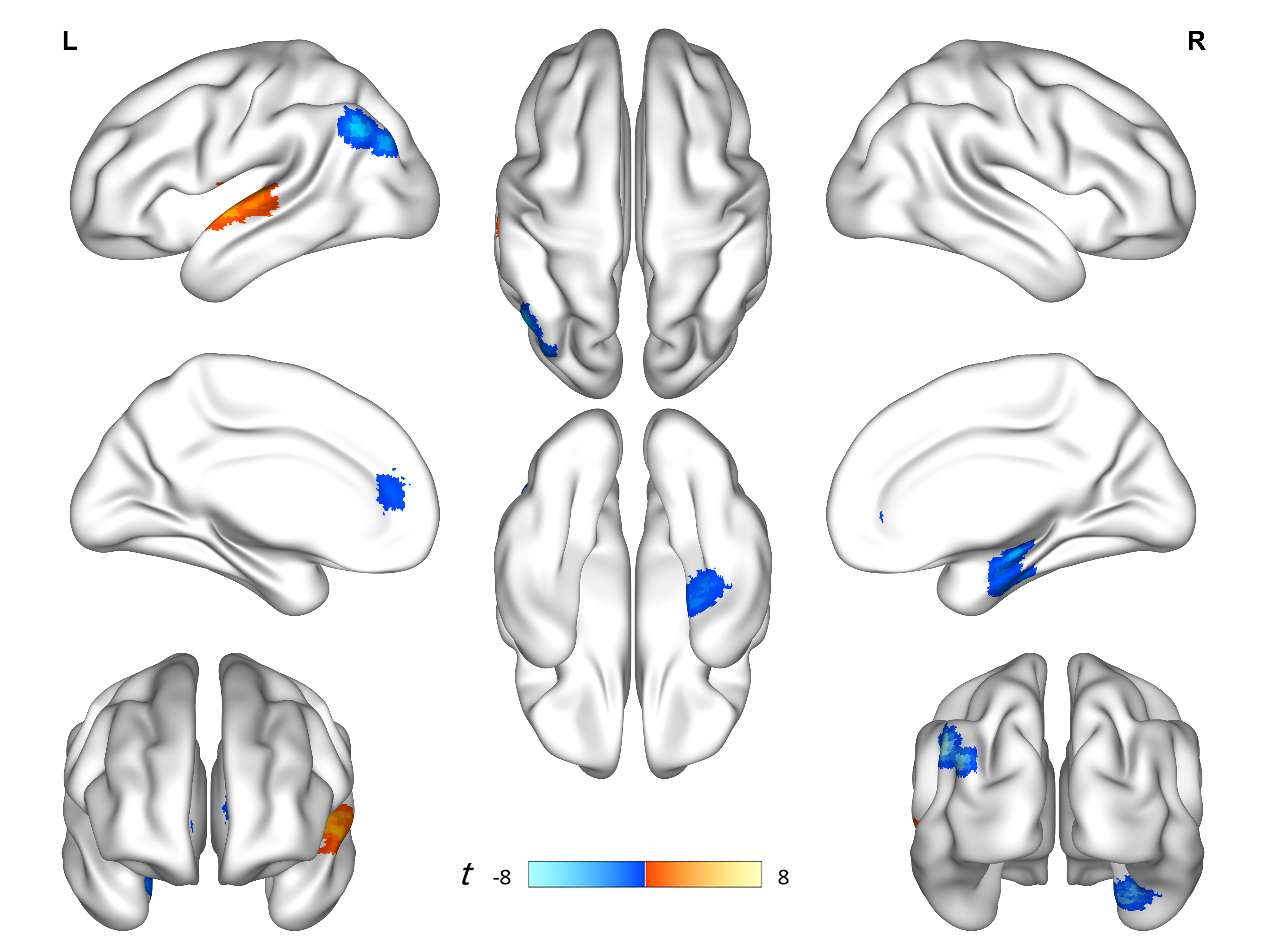


**Fig. S4.** Whole brain voxel-wise functional stability comparison between sedation and awake state, which is calculated from a combination of window size = 80 s and sliding step = 4 s. Abbreviations: L, left; R, right.

**Table S1.** Correlations between functional stability and memory test score

| Brain regions | Explicit memory (Awake) | Implicit memory (Awake) | Explicit memory (Sedated) | Implicit memory (Sedated) |
| --- | --- | --- | --- | --- |
| mPFC  (Awake)  mPFC  (Sedated)  L-AG  (Awake)  L-AG  (Sedated)  Hippocampus  (Awake)  Hippocampus  (Sedated)  L-STG  (Awake)  L-STG  (Sedated) | 0.158 (0.560)  -0.017 (0.951)  -0.0.95 (0.727)  -0.198 (0.463) | -0.223 (0.406)  -0.202 (0.453)  -0.410 (0.115)  0.248 (0.426) | 0.214 (0.426)  -0.080 (0.768)  0.102 (0.707)  -0.187 (0.488) | 0.084 (0.757)  -0.166 (0.539)  0.008 (0.978)  -0.163 (0.546) |

The data are shown as the partial correlation coefficient (*P* value).

Abbreviations: mPFC, medial prefrontal cortex; AG, angular gyrus; STG, superior temporal gyrus
